# Fasciolopsis buski

**DOI:** 10.3201/eid3207.241403

**Published:** 2026-07

**Authors:** Michele Calatri

**Affiliations:** University of Cagliari Faculty of Medicine and Surgery, Cagliari, Italy

**Keywords:** Fasciolopsis buski, fasciolopsiasis, parasites, zoonoses, food safety, etymology, foodborne trematodiasis

## [fas″-ē-ō-lōp′sis buh′-skī]

**Figure 1:**
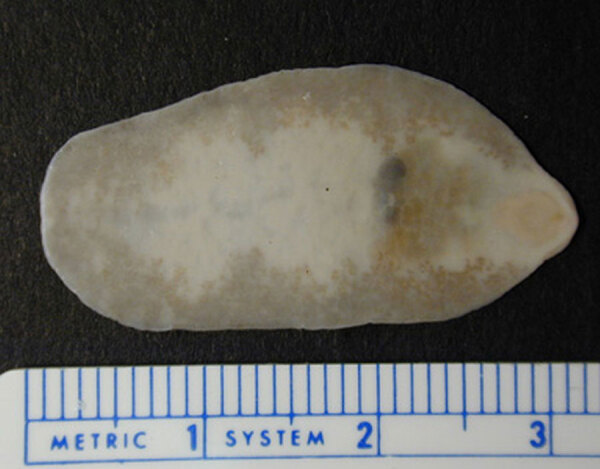
Adult *Fasciolopsis buski* fluke. Source: DPDx (https://www.cdc.gov/dpdx).

**Figure 2:**
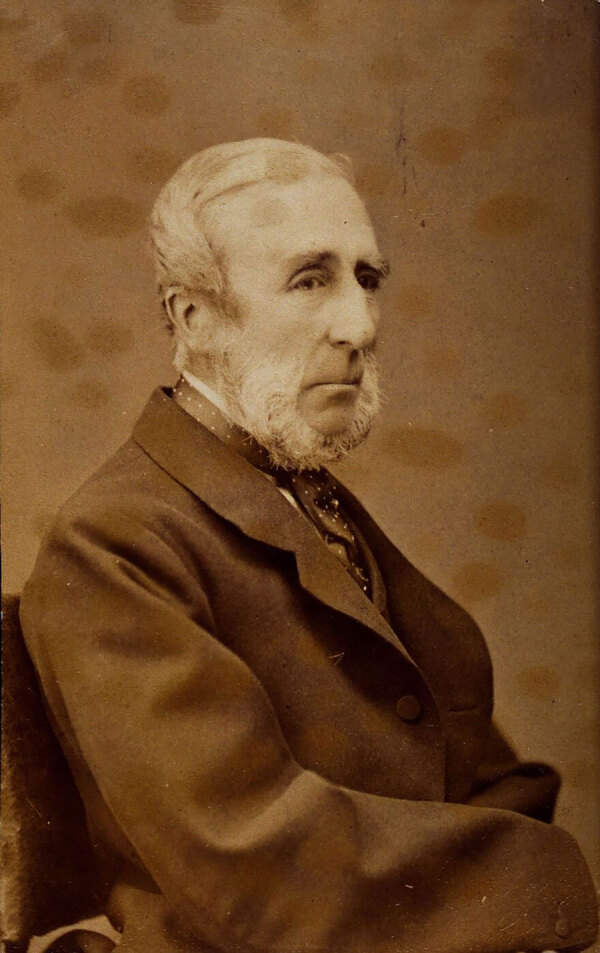
Photograph of George Busk, for whom *Fasciolopsis buski* is named. Source: Wellcome Library, London, UK.

The intestinal fluke *Fasciolopsis buski* is the largest trematode that parasitizes humans, reaching up to 75 mm in length (Figure 1). The genus *Fasciolopsis* was established by Arthur Looss in 1899 and the term derived from the Latin word *fasciola*, meaning small band, and the Ancient Greek suffix *-ὄψῐς* (-opsis), meaning resemblance, referring to the similarity with the members of the genus *Fasciola*. The species was named for the English surgeon George Busk (Figure 2), who identified the adult worm in 1852 from the duodenum of a sailor from India. The first comprehensive description of the species was provided by Edwin Lankester in 1857, and the parasite’s lifecycle was definitively clarified by Koan Nakagawa in 1921.

Fasciolopsiasis is a foodborne trematodiasis that is endemic in rural areas of South and Southeast Asia. The parasite affects both humans and pigs, and pigs act as the main zoonotic reservoir for the parasite. The cercariae, released into the water by various species of planorbid snails, encyst on underwater vegetation. Infection occurs via ingestion of metacercariae on the surface of the water or encysted on freshwater edible plants.
